# Determinants of modern contraceptive utilization among married women of reproductive age group in North Shoa Zone, Amhara Region, Ethiopia

**DOI:** 10.1186/1742-4755-11-13

**Published:** 2014-02-03

**Authors:** Abdurahman Mohammed, Desalegn Woldeyohannes, Amsalu Feleke, Berihun Megabiaw

**Affiliations:** 1 Department of Nursing, College of Medicine and Health Sciences, Debre Birhan University, Debre Birhan, Ethiopia; 2 Department of Health Service Management, Institute of Public Health, College of Medicine and Health Sciences, University of Gondar, Gondar, Ethiopia; 3 Department of Public Health, School of Medicine and Health Sciences, Addis Ababa Science and Technology University, Addis Ababa, Ethiopia; 4 Department of Epidemiology and Biostatistics, Institute of Public Health, College of Medicine and Health Sciences, University of Gondar, Gondar, Ethiopia

**Keywords:** Modern contraceptives, Contraceptive use, Determinants, Ethiopia

## Abstract

**Background:**

Ethiopia is the second most populous country in Africa with high fertility and fast population growth rate. It is also one of the countries with high maternal and child mortality rate in sub-Saharan Africa Family planning is a crucial strategy to halt the fast population growth, to reduce child mortality and improve maternal health (Millennium Development Goal 4 and 5). Therefore, this study aimed to assess the prevalence and determinants of modern contraceptive utilization among married women of reproductive age group.

**Methods:**

A community based cross-sectional study was conducted from August 15 to September 1, 2010 among married women aged 15–49 years in Debre Birhan District. Multistage sampling technique was used to select a total of 851 study participants. A pre-tested structured questionnaire was used for gathering data. Bivariate and multivariate logistic regression analyses were performed using SPSS version 16.0 statistical package.

**Results:**

Modern contraceptive prevalence rate among currently married women was 46.9%. Injectable contraceptives were the most frequently used methods (62.9%), followed by intrauterine device (16.8%), pills (14%), norplant (4.3%), male condom (1.2%) and female sterilization (0.8%). Multiple logistic regression model revealed that the need for more children (AOR 9.27, 95% CI 5.43-15.84), husband approve (AOR 2.82, 95% CI 1.67-4.80), couple’s discussion about family planning issues (AOR 7.32, 95% CI 3.60-14.86). Similarly, monthly family income and number of living children were significantly associated with the use of modern contraceptives.

**Conclusion:**

Modern contraceptive use was high in the district. Couple’s discussion and husband approval of contraceptives use were significantly associated with the use of modern contraceptives. Therefore, district health office and concerned stakeholders should focus on couples to encourage communication and male involvement for family planning.

## Background

In 2011, world population stood at 7 billion. Africa accounts more than 1 billion of world population of which, Ethiopia, the second populous country in Africa contributes 87.1 million people [1]. The average total fertility rate worldwide ranges from 1.7 children per woman in more developed countries to 4.6 in the least developed countries. Total fertility rate in Ethiopia is 5.3 children per woman [1]. This puts Ethiopia among countries with highest total fertility rates in the world. For fertilities to fall to those low levels, increases the use of family planning methods plays a significant contribution especially in less developed countries including Ethiopia [1,2].

At present, family planning service which is free of cost is provided in both governmental and NGO health facilities in Ethiopia, including hospitals, clinics, health centers, and health stations [3]. But , Ethiopia is among countries with low contraceptive prevalence rate, with only 14% and 16%, national and Amhara National Regional State contraceptive prevalence rate among married women, respectively [2,4]. This was resulted high total fertility rate and unwanted pregnancy which intern affects the maternal and child health status [5].

The factors that influence contraceptive practice are multifaceted and challenging. Several studies evident that most women’s knowledge and use of contraception is associated with socio-demographic, socio-cultural, socio economic, source of information and family planning factors. For instance, according to different study findings socio-demographic and economic, obstetric and media exposure related factors were found to contribute on the use of modern contraceptive [6-24].

Considering the present lower utilization of contraceptives achieving the MDGs will be a major challenge for Ethiopia. Therefore, the identification of the prevalence and possible factors that determine the utilization of contraceptives in the study area will have greater input to program managers for designing programs, proper implementation and evaluation of their contribution regarding family planning.

## Methods

### Study design and setting

A community based cross-sectional study was conducted from August 15 to September 1, 2010 in Debre Birhan district, North Shoa Administrative Zone, Amhara National Regional State (ANRS), Ethiopia. The district has 9 administrative kebeles. The district/Debre Birhan town is located at 130 km North of Addis Ababa, capital city of Ethiopia. Based on the 2007 population and housing census, the total population size of the district estimated to be 72,097 [19]. The number of households in the district was estimated to be 16,767 at the time of the study. According to the information obtained from District Health Office; in the district there are 1 hospital, 1 health center, 18 clinics and 4 health posts which render health services for the community. Family planning service is available in most of the health facilities including health posts.

### Sample size and sampling techniques

The source population was all currently married women found in the district, and the study population was currently married women who live in the selected kebeles of each stratum. Currently married women age 15 to 49 years in selected kebeles in each stratum were included.

Assuming 29% modern CPR, 95% confidence level, 4.5% margin of error and design effect of 2, the total sample size required was determined to be 869. A multi-stage sampling technique was employed for the selection of the sampling units. In the district there were 9 kebeles, of which a total of 5 kebeles were selected using simple random sampling method.

The sample size for each of the selected kebeles was determined proportionally to the size of the women age 15–49 years of each kebele. Then, systematic sampling method was employed to select the households from each kebele. The first household interviewed was determined from the kebele house number register using simple random sampling method. The next household was identified systematically (H/h)^th^ by going in a clockwise direction. In cases of selected household with more than one eligible respondent, only one respondent was chosen by lottery method.

### Data collection procedures

Data were collected on utilization of modern contraceptive (female sterilization, male sterilization, the pill, the intrauterine device (IUD), injectables, implants, male condom, female condom, and diaphragm/foam/jelly) and traditional methods (rhythm (periodic abstinence), withdrawal, and folk), socio-demographic characteristics, reproductive and FP factors, and Programmatic factors.

First, a structured and pre-tested questionnaire was prepared in English and then translated into Amharic, the Local language. A questionnaire interview, which was prepared in the Amharic language, was used for data collection. The data was collected by 5 female diploma nurses. The data collectors were supervised daily by two supervisors. The filled questionnaires were checked daily by the supervisors and principal investigator.

Data collectors approached and interviewed the selected respondents after informed consent had been obtained. The women, who were not available in the first visit, were revisited for two more times. During the revisit, the women in the next household were interviewed in place of those women who were not found.

### Data processing and analysis

The data gathered through the structured questionnaire were entered and analyzed using SPSS version 16. Besides, the data were checked and cleaned for their completeness and errors in data entering. Frequency was run and double data entry on 10% of the questionnaires was performed to check data entry errors. To explain the study population in relation to the relevant variables, descriptive statistics were used. Association between dependent and independent variables was assessed and its strength was presented using odds ratios and 95% confidence intervals. Bivariate followed by multivariate logistic regression analysis were also carried out to control confounding effects of variables.

### Ethical consideration

Primarily, ethical clearance was obtained from the Institutional Review Board of the University of Gondar, School of Public Health. Later on, formal letter of cooperation was written for District Health Office. Consent from District Health Office and respective kebeles was also obtained. The data collectors collected the information after obtaining verbal consent from each participant. Respondents were informed that they could refuse or discontinue participation at any time they wanted and they were also informed that they could ask anything about the study. Information was recorded anonymously and confidentiality was assured throughout the study period. A woman who did not practice was advised about the benefits of contraceptive usage and encouraged to communicate with health workers in the district for some more information on it.

## Results

A. Socio-demographic characteristics of the respondents

A total of 851 currently married women responded to the questionnaire, which yields a response rate of 97.9%. Six hundred fifty two (76.6%) of the respondents were from urban areas and the remaining 199 (23.4%) were from the rural areas. The mean age of the respondents was 29.49 years (29.49 ± 6.57 SD). Concerning educational status, 190 (22.3%) of the respondents were unable to read and write, 77 (9%) and 100 (11.8%) of the women and husbands were able to read and write, respectively. Two hundred eighty (32.9%) of the women had attended primary education, 225 (26.6%), were educated to the level of secondary education and the rest 79 (9.3%), attended to college or university level (Table 1).

**Table 1:** Socio-demographic and economic characteristics of currently married women in Debre Birhan District, August 2010

***Variables***		***Frequency (N = 851)***	***Percent (%)***
**Residence**	Urban	652	76.6
	Rural	199	23.4
**Age**	15-19	21	2.5
	20-24	174	20.4
	25-29	273	32.1
	30-34	159	18.7
	35-39	145	17.0
	40-44	62	7.3
	45-49	17	2.0
**Ethnicity**	Amhara	787	92.5
	Oromo	38	4.2
	Others	28	3.3
**Religion**	Orthodox	796	93.5
	Muslim	27	3.2
	Others	28	3.3
**Women educational status**			
Can’t read & write		190	22.3
Read and write only		77	9.0
Primary school (1–8)		280	32.9
Secondary (9–12)		225	26.4
12^+1^ and above		79	9.3
**Husband educational status**			
Can’t read & write		96	11.3
Read and write only		100	11.8
Primary school(1–8)		278	32.7
Secondary(9–12)		226	26.6
12^+1^ and above		151	17.7
**Occupation status of the women**			
House wife/house work		495	58.2
Merchant		89	10.5
Farmer		53	6.2
Daily laborer		113	13.3
Government employee		71	8.3
Others		30	3.5
**Occupation status of the husband**			
Farmer		185	21.7
Merchant		123	14.5
Daily laborer		208	24.4
Government employee		246	28.8
Others		89	10.5
**Monthly family income quartiles**			
≤ 300		213	25
301-600		289	34
601-927		137	16.1
≥ 928		212	24.9
**Having radio/TV status**			
Radio only		293	34.4
TV only		80	9.4
Both radio and TV		324	38.1
None of them		154	18.1

B. Women’s knowledge and practice of modern contraceptive methods

**Figure 1:**
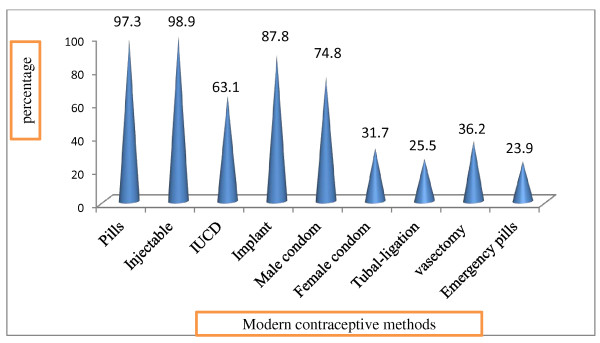
Bar graph showing respondent’s Knowledge of each of modern contraceptive methods in Debre Birhan District, August 2010.

Eight hundred thirty eight (98.5%) of the respondents heard about family planning and were able to mention at least one method. The most commonly reported modern family planning methods were Injectable 815 (98.9%). Regarding the advantages, 591 (70.5%) and 407 (48.6%) of women reported that contraceptive methods have advantage of spacing children and limiting the number of children respectively (Figure 1 and Table 2).

**Table 2:** Knowledge and practice of modern family planning methods among currently married women in Debre Birhan District, August 2010

***Variables***	***Frequency (N)***	***Percent (%)***
**Ever heard about modern contraceptive methods**		
Yes	838	98.5
No	13	1.5
**Source of information**		
HEWs	169	20.7
Radio	481	57.4
Television	460	55.08
Health centers	560	66.83
**Knowledge on advantages of modern contraceptive methods**		
Prevention of unwanted pregnancy	528	63.3
Spacing	591	70.5
Limiting	407	48.6
Preventing STD	103	12.3
**Ever use of modern contraceptive methods (n = 851)**		
Yes	448	52.6
No	403	47.4
**Current use of modern contraceptive methods (n = 851)**		
Yes	399	46.9
No	452	52.1
**Modern contraceptive methods currently being used (n = 399)**		
Pills	56	14.0
Implant/Norplant	17	4.3
Injectable/Depo-Provera	251	62.9
Intrauterine device	67	16.8
Tubal legation	3	0.8
Male condom	5	1.2
**Purpose of using of modern contraceptive (n = 399)**		
Spacing	229	57.4
Limiting	172	42.6

In this study, 448 (52.6%) of women had ever used family planning methods. Three hundred ninety nine (46.9%) [317/652 (48.62%) urban and 82/199 (41.21%) rural] of married women were currently using modern family planning methods. Concerning the types of methods, majority of the respondents were using injectable which is 251 (62.9%) (Table 2).

**Figure 2:**
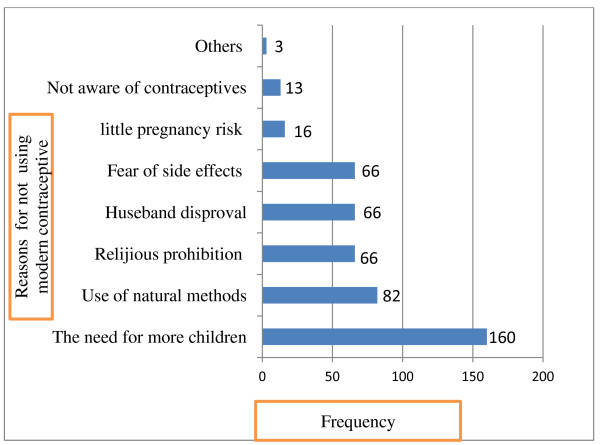
Bar graphs showing reasons for not use or discontinuation of modern contraceptive among currently married women in Debre Birhan District, August 2010.

Among women who had ever used contraceptives, 49 (10.94%) discontinued taking contraceptives. Study participants were forwarded many different reasons for discontinuing or never using modern contraceptives. One hundred sixty (35.5%) need to have more children, 82 (9.6%) due to use of traditional methods, 56 (12.4%) were due to religious prohibition, 56 (12.4%) husband disapproval, 66 (14.6%) fear or/and perceived side effects of contraceptives, like being infertile and developing medical illness such as hypertension were the commonest side effects which perceived by this study participants, 16 (3.9%) due to little pregnancy risk related with being older reproductive age group (45–49) and 13 (2.9%) were related to low awareness about modern family planning (Figure 2).

C. Determinants of modern contraceptive use among currently married women in the district

In the bivariate analysis, educational status of women and husband, desire for more children, number of living children, discussion with health extension workers, discussion of wives with their husband, approval of modern contraceptive use by husband and status of decision making on modern contraceptives were found to be significantly associated with the use of contraceptives with P-value <0.01.

Multivariate analysis was employed to assess the net effect of selected socio-demographic, reproductive, family planning knowledge, perception and program related explanatory variables on the use of modern contraceptive methods. The result of multiple logistic regression model revealed that respondents desire for more children, number of living children, husband approval of use of modern contraceptive methods, husband-wife communication and family monthly income were significantly associated with use of contraceptives methods.

Women who desire another children after two years were 5.71 times more likely to use modern contraceptive than those who desire another child within two years (AOR 5.71, 95% C.I = 3.48-9.37) and those women who do not desire more children at all were 9.27 times more likely to use modern contraceptive methods than those who desire another child within two years (AOR 9.27, 95% C.I. = 5.43-15.84). In addition, those women who perceive/report their husband approve or accept their use of modern contraceptive were almost three times more likely to use modern contraceptive than those who perceive/report their husband would disprove/not accept the use of modern contraceptives (AOR 2.82, 95% C.I 1.67-4.80).

This study also showed that as the number of husband–wife communication increase the use of modern contraceptive also increased when compared with those women who did not communicate with their husband. Those women who had discussion with their husband once were 4.50 times more likely to use modern contraceptives than those women not discussed (AOR 4.50, 95% C.I 2.15–9.42), and those women discussed more than three times were 7.32 times more likely to use modern contraceptives than those women not discussed with their husbands at all about family planning use (AOR 7.32, 95% C.I 3.60–14.86).

Furthermore, the study revealed that family monthly income was significant associated with the use of modern contraceptives. But variables like educational status of the women and husband, residence, women and husband occupation, discussion with health extension workers and some others which are significant or/and P-value < 0.2 in bivariate analysis were not statistically significant with the use of modern contraceptives in the multiple logistic regression model (Table 3).

**Table 3:** Factors associated with the use of modern contraceptives among currently married women of reproductive age group in Debre Birhan District, August 2010

***Explanatory variables***	***Modern***		***COR***	
	***Contraceptive use***		***(95% CI)***	***AOR 95% CI***
	**Yes**	**No**		
**Desire for more children**				
Want within two years	35	166	1	1
Want after two years	161	136	5.62 (3.65-8.63)	5.71 (3.48 - 9.37)***
Want no more	203	150	6.42 (4.21-9.78)	9.27 (5.43-15.84)***
**Number of living children**				
None	30	67	1	1
1-2	206	218	2.11 (1.32-3.38)	1.132 (0.627-2.043)
3-5	150	137	2.45 (1.50-3.99)	1.438 (0.744-2.777)
>5	13	30	0.97 (0.44-2.11)	0.355 (0.131-0.968)*
**Husband approval of the use of modern contraceptive methods**				
Approve	353	232	7.16(4.84-10.59)	2.82 (1.67- 4.80)***
Don’t know	9	46	0.92 (0.42-2.04)	2.50 (0.97-6.44)
Disprove	37	174	1	1
**Family monthly income quartiles**				
≤300	97	116	0.59 (0.40-0.87)	0.66 (0.38-1.12)
301-600	115	174	0.47(0.33-0.67)	0.54 (0.33-0.88)*
601-927	63	74	0.60 (0.39-0.93)	0.50 (0.29-0.85)*
≥928	124	88	1	1
**Discussion with the husband**				
Not discussed	22	181	1	1
Once	80	83	7.90 (4.63-13.59)	4.50 (2.15-9.42)***
Twice	24	32	6.17 (3.10-12.30)	4.10 (1.70-9.92)**
Three times	54	35	12.69 (6.87-23.45)	5.99 (2.65-13.56)***
More than three times	219	121	14.89 (9.08-24.43)	7.32 (3.60-14.86)***

^*^P-value < 0.05, ^**^P-value < 0.01, ^***^P-value < 0.001.

Back ward stepwise multiple logistic regression was used to assess the independent effect of explanatory variables.

## Discussion

This study assessed the prevalence and determinants of modern contraceptive utilization among married women of reproductive age in Debre Birhan district. Nearly half (46.9%) of participants were currently using modern contraceptive methods. This finding was similar with the study done in Adama Town (47%) and also the 51.6% CPR in Ethiopia [20,21]. But this finding is higher than the previous studies conducted in Dembia District, Northwest Ethiopia, in 2004 which showed 14% CPR [15]. The reason for this could be the fact that 76.6% of the study participants were from urban areas in this study while only 41% from the study in Dembia [15]. In addition, increase in the availability and accessibility of different family planning services in both urban and rural areas of Debre Birhan district with active involvement of health extension worker on provision of FP education and service might have increased the use of contraceptive methods.

Most of the study participants were using injectable type of contraceptive methods. This choice could be due to its convenience of not being taken on daily basis and having comfortable ways of administration on top of the availability of this method than others. Child spacing was the main reason for women to use modern contraceptives. On the other hand the need for more children was the main reason for not using modern contraceptive methods. This is in line with findings from northwest Ethiopia and Sudan, Bangladesh and Pakistan [6,10,12,14,15].

In this study, those women who desire another child after two years were nearly six times more likely to use modern contraceptives than those women who desire another child within two years. This finding is in line with the study conducted in Bangladesh and Pakistan [6,16]. Women’s internal motivations to achieve their child spacing goal could be the possible reason for higher level of contraceptive use.

Women who had one or more children were more likely to use modern contraceptives than those who had no children. In contrast, women who had more than 5 children were almost three times less likely to use modern contraceptives compared with women who had no children. This could be for the reason that those women who had more than 5 children were at the older age of reproduction and they might have perceived not to be at risk of pregnancy. Secondly, older women are more likely to practice sex infrequently. This finding is similar to previous reports from Bangladesh, Pakistan, and Tanzania which reported that as the number of living children increases, use of modern contraceptives increase [6,8,16].

This study revealed that women who discussed with their husband about modern contraceptives were about seven times more likely to use modern contraceptive methods than women who did not discuss at all. This is in line with studies conducted from Jimma (south west Ethiopia), Kenya, Tanzania, Nigeria and Bangladesh [6,8,9,13,14,17].

This study also indicated that those women whose husband’s approve using modern contraceptives were almost three times more likely to use modern contraceptives. This is in line with the previous studies in Pakistan and Ethiopia [9,15,22,23]. This implies that male involvement has an important role on the use of modern contraceptives.

The main strength of this study is that, being community based, it could reflect the actual experience of the married women during the study period. As limitations of this study, information about the husband were indirectly obtained from women. Additionally, similarities of living standards in some rural and urban areas might have underestimated the rural–urban difference in the use of contraceptive methods. On the other hand, being most of the residences in the district were live in urban areas might not able to identify factors which associate with the use of modern contraceptive in the rural areas.

This study were tried to see the strength of the association between the outcome and explanatory variables beyond simply identifying those variables which are statistically significant for the use of modern contraceptive specifically in the study area.

In conclusion, the modern contraceptive use in this district among currently married women of reproductive age group is high. Couple’s communication/discussion and husband’s approval about contraceptives were significantly associated with modern contraceptive use. However, women’s demand for more children has a negative effect on the use of modern contraceptive methods. Improvement in family income or socio-economic status to the higher level has an important role in upgrading usage of modern contraceptive.

Health service programs and strategies of the country at each level of health care delivery system need to consider the involvement of males for modern contraceptives utilization. Hence, governmental and nongovernmental organizations, health facilities and other stakeholders need to ensure availability, accessibility and sustained advocacy for use of available contraceptive methods for married couples. Lastly, we recommend that researchers to investigate husbands’ perception and acceptance toward contraceptive use by their couples.

## Abbreviations

ANRS: Amhara National Regional State; CPR: Contraceptive prevalence rate; EDHS: Ethiopian demographic health survey; FGAE: Family guidance association of ethiopia; FP: Family planning; HEW: Health extension worker; IUCD: Intrauterine contraceptive device; MDG: Millennium development goal; NGO: Non-governmental organization; RH: Reproductive health; SD: Standard deviation; WHO: World Health Organization.

## Competing interests

The authors declare that they have no competing interests.

## Authors’ contributions

AM designed the study, participated in the data collection, performed analysis and interpretation of data and drafted the paper and prepared the manuscript. BM, AF and DW assisted in the design, approved the proposal with some revisions, participated in data analysis and revised subsequent drafts of the manuscript. All authors read and approved the final manuscript.
